# Evaluation of Neutrophil/Lymphocyte Ratio, Low-Density Lipoprotein/Albumin Ratio, and Red Cell Distribution Width/Albumin Ratio in the Estimation of Proteinuria in Uncontrolled Diabetic Patients

**DOI:** 10.7759/cureus.44497

**Published:** 2023-08-31

**Authors:** Duygu Tutan, Murat Doğan

**Affiliations:** 1 Department of Internal Medicine, Erol Olçok Training and Research Hospital, Çorum, TUR

**Keywords:** diabetic nephropathy, inflammation, proteinuria, end-stage renal disease, diabetes

## Abstract

Introduction

Diabetic nephropathy associated with end-stage renal disease (ESRD) is a significant public health problem due to its high morbidity and mortality. We aimed to improve the estimation of proteinuria in diabetic patients and potentially enhance risk stratification and clinical management strategies with the assessment of the correlation of the neutrophil/lymphocyte ratio (NLR), low-density lipoprotein/albumin ratio (LAR), and red cell distribution width/albumin ratio (RAR) with the proteinuria in the uncontrolled diabetes patient population.

Methods

This was a retrospective study including 327 patients with uncontrolled diabetes (HbA1c > 10%) seen in an outpatient clinic. The study enrolled patients over 18 years old, excluding those with active infections, malignancies, immunodeficiency, hematological diseases, pregnancy, breastfeeding, or type I diabetes. Patients using specific drugs affecting proteinuria or lipid levels were also excluded. Data from patients were retrospectively obtained, including gender, age, blood parameters, glucose, creatinine, albumin, cholesterol, triglyceride, and HbA1c levels, as well as spot urine protein and creatinine levels. Proteinuria was assessed using a spot urine protein/creatinine ratio (>0.30 indicated proteinuria). Patients were divided into two groups: group 1 (non-proteinuric with uncontrolled diabetes) and group 2 (proteinuric with uncontrolled diabetes). Demographics, laboratory results, and LAR, NLR, and RAR values were compared between the groups with univariate and multivariate analyses. All statistical analyses were performed using IBM SPSS Statistics for Windows software. For the statistical significance level, p<0.05 was accepted as meaningful.

Results

Among 327 patients with uncontrolled diabetes, 33.03% were proteinuric. Patients with proteinuria were significantly older (median age 65 vs. 61 years) and had higher NLR and RAR values. There were no significant differences observed in terms of LAR values between groups. Serum albumin levels were lower and urea levels were higher in the proteinuric group. A multivariate analysis was done to identify variables for the prediction of proteinuria. NLR and RAR were found to be independent predictors of proteinuria even after adjusting for potential confounders in the multivariate analysis. The model achieved a 71.9% correct classification rate. An NLR cutoff of 1.93 increased the likelihood of proteinuria 1.93-fold, while a RAR cutoff of 3.30 increased the likelihood 1.63-fold.

Conclusions

We found that the LAR ratio cannot be used to predict proteinuria in patients with HbA1C levels above 10, but the NLR and RAR ratios can guide the clinician regarding proteinuria and potentially enhance risk stratification and clinical management strategies before a detailed workup.

## Introduction

Diabetes mellitus (DM) is an important metabolic disorder characterized by elevated blood glucose concentrations, and it is increasing in frequency [1]. Patients with uncontrolled diabetes impose a significant financial burden on families, health systems, and countries [2]. Patients with poor glycemic control are also at increased risk of microvascular complications. It is reported in the literature that the risk of nephropathy increases nonlinearly as the hemoglobin A1c (HbA1c) level increases above 10% [3].

Diabetic nephropathy is a common microvascular complication of type 2 diabetes and occurs in 40% of patients [4]. It is characterized by the presence of albuminuria and a decline in renal function. The natural course of diabetic kidney disease progresses as glomerular hyperfiltration, progressive albuminuria, proteinuria, decreased glomerular filtration rate (GFR), and finally, end-stage renal disease (ESRD) [5]. This process is complex and involves various mechanisms, including inflammation, oxidative stress, endothelial dysfunction, glomerular basement membrane thickening, and fibrosis [6].

Chronic inflammation, oxidative stress, and inflammation play significant roles in the progression of diabetic proteinuria. Elevated blood glucose levels promote the release of pro-inflammatory cytokines and chemokines, leading to the infiltration of immune cells, such as macrophages, into the kidney and contributing to the disruption of the glomerular filtration barrier and the development of proteinuria [7]. These inflammatory cells release reactive oxygen species (ROS) and other damaging molecules, which further contribute to renal injury and proteinuria. ROS can cause damage to the glomerular endothelial glycocalyx, a protective layer that contributes to the glomerular barrier function [8]. Increased oxidative stress in the kidney can lead to podocyte injury and dysfunction, contributing to the development of proteinuria [8]. Inflammatory markers, such as C-reactive protein (CRP), have been associated with diabetic nephropathy and proteinuria [9].

Diabetic nephropathy associated with ESRD is a significant public health problem due to its high morbidity and mortality. It is also a common and significant complication of uncontrolled diabetes, serving as a crucial indicator of renal dysfunction and an independent risk factor for the progression of diabetic nephropathy [10]. However, current methods for estimating proteinuria, such as urine protein dipstick tests and quantitative urine protein measurements, have limitations in terms of accuracy and reliability. As a result, there is a growing interest in identifying novel markers that can provide a more comprehensive assessment of proteinuria in diabetic patients [11].

In this study, we sought to evaluate the potential utility of three ratios, namely neutrophil/lymphocyte ratio (NLR), low-density lipoprotein (LDL)/albumin ratio (LAR), and red blood cell distribution width (RDW)/albumin ratio (RAR), as cheap, easily accessible, noninvasive markers for proteinuria in uncontrolled diabetic patients to detect diabetic nephropathy-related proteinuria at an early stage. By exploring these ratios, we aim to improve the estimation of proteinuria in diabetic patients and potentially enhance risk stratification and clinical management strategies.

## Materials and methods

This study was planned as a retrospective study and approved by the Hitit University Faculty of Medicine Ethics Committee (Decision No. 2023-89, Date: July 26, 2023). All patients who were seen at the Erol Olçok Training and Research Hospital, Department of Medicine outpatient clinic with uncontrolled diabetes (HbA1c > 10%) between January 1st, 2021, and March 31st, 2023, were screened retrospectively. Patients over 18 years of age without any acute or chronic active infections, active malignancy, immunodeficiency, a known hematological disease, pregnancy, or breastfeeding status were included, and patients with type I diabetes mellitus, patients who are using drugs that can exacerbate proteinuria or change lipid levels/proteinuria (statins, ACE inhibitors, SGLT-2 inhibitors, etc.), patients using drugs that alter hemogram parameters (steroids, cytotoxic agents, etc.), and those whose blood results could not be obtained, were excluded. A total of 327 diabetes mellitus type II patients were included in the study. The gender, age, serum neutrophil, lymphocyte, monocyte, platelet counts, RDW, hemoglobin, fasting glucose, creatinine, urea, albumin, LDL cholesterol, triglyceride, HbA1c levels, spot urine protein, and spot urine creatinine levels of 327 patients were obtained retrospectively from the archive system. Proteinuria was assessed using the spot urine protein/spot urine creatinine ratio. Patients with a ratio exceeding 0.30 were categorized as having proteinuria. All patients were divided into two groups: non-proteinuric patients with uncontrolled diabetes as group 1 and proteinuric patients with uncontrolled diabetes as group 2. These groups were compared in terms of demographic characteristics, laboratory results, and indicators such as LAR, NLR, and RAR. The LAR score was calculated as LAR = LDL cholesterol/serum albumin level, the NLR score was calculated as NLR = neutrophil count/lymphocyte count, and the RAR score was calculated as RAR = RDW/serum albumin level.

All statistical analyses were performed using IBM SPSS Statistics for Windows software (Version 26; IBM Corp., Armonk, NY, USA). Descriptive statistics were reported using numbers and percentages for categorical variables. Numerical variables were reported as mean ± standard deviation for Gaussian distributed variables and median value accompanied by minimum and maximum values in parentheses for non-Gaussian distributed variables. Data distribution was evaluated using the Shapiro-Wilks test. Relationships between variables were investigated with Pearson and Spearman correlation coefficients. The comparison of numerical measurements for two independent research groups was made according to distribution. Age, serum neutrophil, lymphocyte, monocyte, platelet counts, RDW, glucose, albumin, creatinine, urea, triglyceride, HbA1c levels, spot urine creatinine, spot urea protein, and LAR, NLR, and RAR were evaluated with the Mann-Whitney U test, and serum hemoglobin level and LDL cholesterol level were assessed using the student t-test. The chi-square test was used to evaluate the statistical significance of categorical variable differences across groups. A binomial logistic regression test was used for the multivariate analysis, including gender, age, HbA1c levels, LAR, NLR, and RAR, to determine the association between the variables and the existence of proteinuria after adjusting for confounding factors. By drawing the ROC curve and determining the area under it, the optimal LAR, NLR, and RAR cut-off values with the best sensitivity and specificity that separate the groups based on proteinuria were calculated using the Youden index. Sensitivity, specificity, PPV, NPV, test precision, and odds ratio were calculated for cut-off values. For the statistical significance level, p<0.05 was accepted as meaningful.

## Results

A total of 327 patients who were seen at the outpatient clinic were included in the study. 148 of the patients (45.26%) were male, and 179 (54.74%) were female. The median age of all patients was 61 years, the youngest patient was 23 years old, and the oldest patient was 92 years old. The median HbA1c was 11.2% (10-18.2%). Of 327 patients, 219 had no proteinuria (66.97%), and 108 had proteinuria (33.03%).

In the comparative analysis between the two groups, no significant difference was observed between gender distribution, monocyte count, platelet count, RDW, hemoglobin, glucose, creatinine, LDL cholesterol, spot urine creatinine, triglyceride, and HbA1c levels (Table 1).

**Table 1:** Evaluation and comparison of the variables between two groups and the results of the univariate and multivariate analyses RDW: red cell distribution width, LDL: low-density lipoprotein, HbA1c: hemoglobin A1c, LAR: low-density lipoprotein to albumin ratio, NLR: neutrophil to lymphocyte ratio, RAR: red cell distribution width to albumin ratio, CI: confidence interval

Variables		All patients (n=327)	Univariate analysis			Multivariate logistic regression analysis		
			Non-proteinuric (n=219)	Proteinuric (n=108)	Statistical significance	Wald	Exp(B) (CI 95%)	Statistical significance
Gender	Male	148 (45.26%)	101 (46.12%)	47 (43.52%)	0.657	0.087	0.925 (0.553–1.549)	0.768
	Female	179 (54.74%)	118 (53.88%)	61 (56.48%)				
Age (years)		61 (23-92)	60 (23-85)	65 (25-92)	0.001	1.943	1.016 (0.994–1.038)	0.163
Hemoglobin (g/dL)		14.7±1.64	14.78±1.54	14.53±1.82	0.231			
Lymphocyte (10^9^/L)		2.51 (0.61–5.39)	2.56 (0.87–5.39)	2.34 (0.61–4.45)	0.008			
Monocyte (10^9^/L)		0.53 (0.08–2.02)	0.53 (0.08–1.55)	0.53 (0.11–2.02)	0.999			
Neutrophil (10^9^/L)		4.25 (1.13–9.77)	4.13 (1.42–8.62)	4.51 (1.13–9.77)	0.012			
Platelet (10^9^/L)		252 (83–590)	253 (83–474)	250.5 (102–590)	0.861			
RDW (%)		12.9 (11.3–18.4)	12.9 (11.3–18.4)	12.95 (11.4–17.9)	0.308			
Glucose (mg/dL)		256 (61–674)	252 (85–545)	268 (61–674)	0.252			
Albumin (g/dL)		4.2 (2.7–4.9)	4.2 (3.3–4.9)	4.05 (2.7–4.8)	<0.001			
Creatinine (mg/dL)		0.7 (0.3–2.7)	0.7 (0.4–1.7)	0.8 (0.3–2.7)	0.058			
Urea (mg/dL)		31 (12–106)	29 (12–106)	33.5 (16–86)	0.001			
LDL cholesterol (mg/dL)		118.43±39.65	118.73±39.1	117.81±40.93	0.843			
Spot urine creatinine (mg/dL)		86.94 (7.92–611.27)	94.18 (16.87–611.27)	64.05 (7.92–197.5)	0.058			
Spot urine protein (mg/dL)		18.93 (1.28–689.83)	14.97 (1.28–62.19)	39.11 (4.73–689.83)	0.001			
Triglyceride (mg/dL)		160 (43–2278)	158 (55–1312)	169 (43–2278)	0.427			
HbA1c (%)		11.2 (10–18.2)	11.2 (10–16.3)	11.3 (10–18.2)	0.461	1.364	1.117 (0.928–1.345)	0.243
Proteinuria level		0.22 (0.05–13.83)	0.16 (0.05–0.3)	0.54 (0.3–13.83)	<0.001			
LAR		28.46 (3.02–80.74)	27.44 (4.55–53.75)	30.38 (3.02–80.74)	0.299	0.231	1.006 (0.982–1.031)	0.631
NLR		1.74 (0.54–7.92)	1.66 (0.54–4.10)	2.00 (0.77–7.92)	<0.001	10.606	1.606 (1.208–2.136)	0.001
RAR		3.09 (2.33–5.30)	3.07 (2.33–4.49)	3.14 (2.60–5.30)	0.002	4.296	1.935 (1.037–3.611)	0.038

The median age of patients with proteinuria was 65 years, which was significantly higher than that of patients without proteinuria (p=0.001). Higher neutrophil and lymphocyte values were observed in the proteinuric group compared to normal patients (p=0.012, p=0.008, respectively). The median serum albumin level of proteinuric patients was 4.05 mg/dL, while the median serum albumin level of normal patients was 4.20 mg/dL, and albumin was significantly lower in the presence of proteinuria (p<0.001). Proteinuric patients had higher urea levels than normal patients (29 mg/dL vs. 33.5 mg/dL, p=0.001). The median value of the spot urine protein in proteinuric patients was found to be higher at 39.11 mg/dL compared to 14.97 mg/dL in normal patients (p=0.001).

There was no significant difference between proteinuric and non-proteinuric patients in terms of the LAR ratio (p=0.299). While the median NLR in the non-proteinuric group was 1.66, this ratio was found to be 2.00 in the proteinuric group and was higher with a statistically significant difference (p<0.001). The RAR ratio was also significantly higher in the proteinuric group (3.07 vs. 3.14, p=0.002).

A multivariate analysis including age, gender, HbA1c, LAR, NLR, and RAR as covariates also showed no significant differences in gender, HbA1c, and LAR values between the groups (p=0.163, p=0.243, and p=0.631, respectively). While being significant in the univariate analysis, age lost its significance in the multivariate analysis (p=0.163, Table 1). The binomial logistic regression model correctly classified 71.9% of the cases (R2 = 0.130, p<0.001), and NLR and RAR remained as independent predictors even after adjusting for potential confounders in the multivariate analysis (Exp(B) with 95% CI = 1.606 (1.208-2.136), p=0.001 for NLR and 1.935 (1.037-3.611), p=0.038 for RAR).

**Figure 1:**
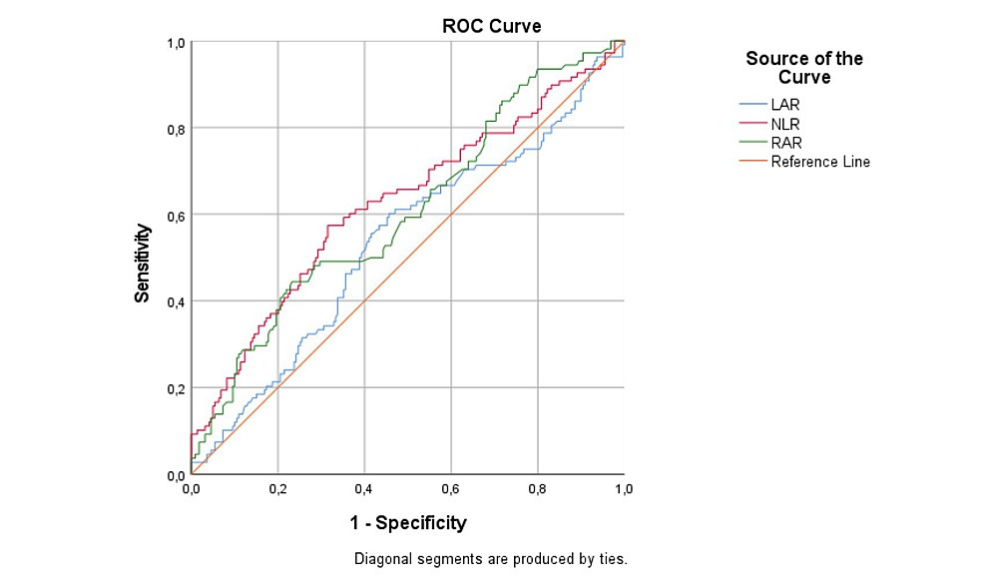
Receiver operating curve of LAR, NLR, and RAR in the prediction of proteinuria in uncontrolled diabetes patients LAR: low-density lipoprotein to albumin ratio, NLR: neutrophil to lymphocyte ratio, RAR: red-cell distribution width to albumin ratio, ROC: receiver operating curve

In the ROC analysis performed to find the optimal values of NLR and RAR for proteinuria discrimination, a value of 1.93 was found optimal for NLR with 57.4% sensitivity, 68.5% specificity, 47.3% PPD, 76.5% NPD, and 64.8% test accuracy, and a value of 3.30 for RAR with 44.4% sensitivity, 76.7% specificity, 48.5% PPD, 73.7% NPD, and 66.1% test accuracy (Figure 1). NLR of 1.93 and above increased the likelihood of proteinuria 1.93-fold, while RAR of 3.30 and above increased the likelihood of proteinuria 1.63-fold (Table 2).

**Table 2:** Diagnostic properties of the cut-off values for LAR, NLR, RAR in the prediction of proteinuria in uncontrolled diabetes patients LAR: low-density lipoprotein to albumin ratio, NLR: neutrophil to lymphocyte ratio, RAR: red cell distribution width to albumin ratio, PPV: positive predictive value, NPV: negative predictive value, ROC: receiver operating curve, SE: standard error, CI: confidence interval, OR: odds ratio

Variables	Cut-off	Diagnostic values					ROC analysis			Odds ratio		
		Sensitivity	Specificity	PPV	NPV	Accuracy	Area (SE)	%95 CI	p	OR	%95 CI	p
LAR	-	-					0.535 (0.035)	0.467-0.603	0.299	-		
NLR	1.93	57.4%	68.5%	47.3%	76.5%	64.8%	0.625 (0.034)	0.558-0.691	<0.001	2.930	1.820–4.717	<0.001
RAR	3.30	44.4%	76.7%	48.5%	73.7%	66.1%	0.605 (0.033)	0.540-0.671	0.002	2.635	1.611–4.311	<0.001

## Discussion

Proteinuria is a common complication of uncontrolled diabetes and is associated with an increased risk of renal dysfunction and cardiovascular disease [12]. In this study, we investigated the correlation between the neutrophil/lymphocyte ratio, low-density lipoprotein/albumin ratio, red cell distribution width/albumin ratio, and proteinuria in uncontrolled diabetic patients. Our findings revealed a significant positive correlation between the neutrophil/lymphocyte ratio and proteinuria, indicating the potential involvement of inflammation and immune imbalance in the development of diabetic proteinuria. However, the low-density lipoprotein/albumin ratio did not exhibit a significant correlation with proteinuria in our study. Despite this, the red cell distribution width/albumin ratio demonstrated a significant positive correlation with proteinuria, suggesting that abnormalities in red blood cell dynamics may contribute to renal dysfunction and subsequent protein leakage.

The significance of the NLR in diabetic nephropathy and other diseases has been previously reported [13]. A study by Jaaban et al. [14] found that NLR was a novel risk marker for diabetic nephropathy in patients with type 2 diabetes. The observed correlation between the neutrophil/lymphocyte ratio and proteinuria supports the notion that inflammation and immune dysregulation play crucial roles in the pathogenesis of diabetic proteinuria. Chronic hyperglycemia in diabetes promotes a pro-inflammatory state, leading to increased neutrophil counts and subsequent tissue inflammation [14]. One of the potential mechanisms underlying the association between NLR and proteinuria could be that this chronic inflammation can contribute to immune dysfunction, altered responses to inflammation, renal damage, and ultimately proteinuria [15,16]. Immune dysfunction, characterized by altered neutrophil and lymphocyte counts, may disrupt the immune response against renal injury, thereby facilitating the development of proteinuria [16]. Regarding the red cell distribution width/albumin ratio, abnormalities in red blood cell dynamics may reflect microvascular and hemodynamic alterations that impact renal function and the integrity of the filtration barrier. Zhang et al. [17] investigated the association between RDW and diabetic nephropathy (DN) in patients with type-2 diabetes mellitus (T2DM) and found that patients with higher RDW levels had more severe glomerular damage, suggesting a potential link between RDW and the progression of DN.

One of the key factors involved in the progression of diabetic nephropathy is chronic subclinical inflammation [18]. Inflammatory markers, such as tumor necrosis factor-α (TNF-α), have been found to be elevated in patients with diabetic nephropathy [18]. Inflammation can lead to endothelial dysfunction, increased vascular permeability, and the recruitment of immune cells, which contribute to the development of renal injury [19]. Inflammatory cytokines and chemokines, such as interleukin-1β (IL-1β) and monocyte chemoattractant protein-1 (MCP-1), are also involved in the recruitment and activation of immune cells in the kidney [19]. Oxidative stress, which is closely linked to chronic inflammation, is another important factor in the progression of diabetic nephropathy [20,21]. Increased production of ROS and reduced antioxidant defenses contribute to oxidative stress in the kidney [20]. Oxidative stress can activate inflammatory pathways and promote the release of pro-inflammatory cytokines, further exacerbating inflammation [21]. Inflammation and oxidative stress can lead to the deposition of extracellular matrix proteins, such as collagen, in the kidney [19,20]. This process, known as fibrosis, contributes to the structural changes observed in diabetic nephropathy, including glomerulosclerosis and tubulointerstitial fibrosis [22]. Fibrosis impairs renal function and can ultimately lead to end-stage renal disease [22].

Evaluating the neutrophil/lymphocyte ratio, low-density lipoprotein/albumin ratio, and red cell distribution width/albumin ratio in estimating proteinuria provides an opportunity to compare them with existing biomarkers. Traditional methods for estimating proteinuria, such as urine protein dipstick tests, have limitations in terms of accuracy and reliability [23]. The positive correlation observed between the neutrophil/lymphocyte ratio and proteinuria suggests that this ratio may provide valuable information regarding the inflammatory state and immune dysregulation associated with proteinuria in diabetes. While the low-density lipoprotein/albumin ratio did not exhibit a significant correlation in our study, it is essential to note that dyslipidemia, particularly elevated LDL levels, remains an important risk factor for the development and progression of diabetic nephropathy [24,25]. The red cell distribution width/albumin ratio, reflecting red blood cell dynamics, demonstrated a significant correlation with proteinuria, indicating its potential as a non-invasive marker. Future studies should further explore the diagnostic accuracy and predictive value of these ratios in comparison to existing biomarkers, providing insights into their potential clinical utility.

Accurate estimation of proteinuria is crucial for risk stratification and clinical management in uncontrolled diabetic patients [26]. Identifying patients with significant proteinuria allows for timely intervention and targeted treatment strategies to slow down the progression of diabetic nephropathy [26]. While the neutrophil/lymphocyte ratio demonstrated a significant correlation with proteinuria in our study, further research is necessary to assess its clinical utility in risk stratification. The absence of a significant correlation between the low-density lipoprotein/albumin ratio and proteinuria in our study suggests that this ratio may not be a reliable marker for proteinuria estimation in uncontrolled diabetic patients. However, given the importance of dyslipidemia in diabetic nephropathy, lipid profile assessment and control remain essential in clinical practice [24]. The significant correlation between the red cell distribution width/albumin ratio and proteinuria indicates its potential as a non-invasive marker for renal dysfunction in diabetes. Incorporating these ratios into routine clinical practice may assist in identifying patients who require closer monitoring, more aggressive glycemic and blood pressure control, and lifestyle interventions. Furthermore, these ratios may aid in identifying patients who would benefit from early referral to nephrologists for specialized care and intervention, ultimately improving patient outcomes and reducing the burden of end-stage renal disease.

Several limitations should be considered when interpreting the findings of this study. First, the study design was cross-sectional, limiting our ability to establish causality or determine the predictive value of the ratios for future renal outcomes. Prospective longitudinal studies are needed to assess the prognostic significance of these ratios and their ability to predict the progression of diabetic nephropathy. Second, the study focused on a specific group of uncontrolled diabetic patients, which may limit the generalizability of the results to other diabetes subgroups or patients with controlled diabetes. Additionally, our study did not explore other potential ratios or markers that may be relevant to proteinuria estimation in diabetic patients. Future research should investigate additional ratios and markers to evaluate their diagnostic accuracy and clinical utility comprehensively.

Despite the limitations mentioned, this study has several strengths. First, the evaluation of multiple ratios provides a comprehensive assessment of potential markers for proteinuria in uncontrolled diabetic patients. Second, the inclusion of a well-defined patient population adds to the validity and reliability of the findings. Lastly, the utilization of appropriate statistical analyses enhances the robustness of the correlations observed between the neutrophil/lymphocyte ratio and proteinuria, as well as the red cell distribution width/albumin ratio and proteinuria.

## Conclusions

In conclusion, our study demonstrated a significant positive correlation between the neutrophil/lymphocyte ratio and proteinuria, suggesting the involvement of inflammation and immune dysregulation in the pathogenesis of diabetic proteinuria. The red cell distribution width/albumin ratio also exhibited a significant positive correlation with proteinuria, indicating potential abnormalities in red blood cell dynamics associated with renal dysfunction. However, the low-density lipoprotein/albumin ratio did not demonstrate a significant correlation with proteinuria in our study. These findings highlight the potential utility of the neutrophil/lymphocyte ratio and the red cell distribution width/albumin ratio as non-invasive markers for proteinuria in uncontrolled diabetic patients, and these ratios can guide the clinician regarding proteinuria before a detailed workup. Further research is warranted to elucidate the underlying mechanisms, compare these ratios with existing biomarkers, and evaluate their clinical utility for risk stratification and management of diabetic nephropathy. Understanding and accurately estimating proteinuria in diabetes is crucial for early intervention, personalized treatment strategies, and improved outcomes in patients with uncontrolled diabetes. The identification of reliable and non-invasive markers for proteinuria holds promise for early detection and intervention, ultimately improving outcomes for individuals with uncontrolled diabetes and reducing the burden of diabetic nephropathy.
